# Understanding side effects of therapy for myasthenia gravis and their impact on daily life

**DOI:** 10.1186/s12883-019-1573-2

**Published:** 2019-12-21

**Authors:** Elizabeth Dansie Bacci, Karin S. Coyne, Jiat-Ling Poon, Linda Harris, Audra N. Boscoe

**Affiliations:** 1 Patient-Centered Research, Evidera, 1417 Fourth Avenue Suite 510, Seattle, WA 98101 USA; 2 Patient-Centered Research, Evidera, Bethesda, MD USA; 3 Patient-Focused Outcomes Center of Expertise, Eli Lilly, Indianapolis, IN USA; 4 Global Health Outcomes Research, Alexion Pharmaceuticals Inc, New Haven, CT USA; 5 Health Economics and Outcomes Research, Agios Pharmaceuticals, Cambridge, MA USA

**Keywords:** Myasthenia gravis, MG, Adherence to treatment, Side effects, Health-related quality of life

## Abstract

**Background:**

Myasthenia gravis is a chronic, autoimmune, neuromuscular junction disorder characterized by skeletal muscle weakness. Current therapies for myasthenia gravis are associated with significant side effects. The objective of this study was to characterize the side effects, and associated health-related quality of life and treatment impacts, of traditional myasthenia gravis treatments.

**Methods:**

This study had two phases; a Phase 1 interview and a 2-part web-based survey in Phase 2 that included brainstorming (Step 1) and rating (Step 2) exercises using group concept mapping. In Phase 1, all 14 participants reported experiencing side effects from myasthenia gravis treatments which had significant impacts on daily life. In Phase 2, 246 participants contributed to Step 1; 158 returned for Step 2.

**Results:**

The brainstorming exercise produced 874 statements about side effects and their impact, which were reduced to 35 side effects and 23 impact-on-daily life statements. When rating these statements on severity, frequency, and tolerability, blood clots, infections/decreased immunity, weight gain, and diarrhea were the least tolerable and most severely rated. The most frequent and severe impacts were sleep interference and reduced physical and social activities.

**Conclusions:**

Based on these findings, there appears to be a need for better and more tolerable treatments for myasthenia gravis patients.

## Background

Myasthenia gravis (MG) is a chronic, autoimmune, neuromuscular junction disorder characterized by varying degrees of weakness of the skeletal muscles of the body [1]. Published prevalence estimates of MG vary widely; the Myasthenia Gravis Foundation of America (MGFA) suggests the prevalence of MG to be approximately 14 to 20 per 100,000, or at least 35,000 to 60,000 individuals in the United States (US) [2]. The presentation of MG includes fluctuating, and fatigable weakness of various muscle groups. This muscle weakness increases during periods of activity and improves after periods of rest. Muscles that control eye and eyelid movement, facial expression, chewing, talking, and swallowing can be affected, in addition to those that control breathing and neck and limb movements [3]

Currently there is no cure for MG, but various traditional therapies, including acetylcholinesterase inhibitors and immunomodulating therapies (corticosteroids, azathioprine, and mycophenolate mofetil) allow most patients to live productive lives with a normal life expectancy. However, these therapies are associated with side effects that can deter patients from their use, such as nausea, vomiting, gastrointestinal (GI) upset, increased risk of infection, weight gain, or liver damage [4]. Given the side effect profile of traditional therapies, new targeted therapies have been developed. The first, eculizumab, a terminal complement inhibitor, was recently approved by the Food and Drug Administration (FDA), European Commission, and The Ministry of Health, Labor, and Welfare in Japan in 2017.

Unsatisfactory treatment outcomes with traditional MG therapies also contribute to poor adherence, with 23% of patients reporting poor treatment compliance [5]. Despite this, information on the precise nature, duration, and severity of these side effects from the patient perspective is scarce. Furthermore, the impact of these side effects on patient health-related quality of life (HRQOL), daily life, and adherence to treatment is not clearly understood.

The objectives of this study were to characterize patient experiences with side effects for traditional MG treatments, and to understand the impact of the side effects on patients’ HRQOL, daily life, and adherence to therapy.

## Materials and methods

### Study design

This study had two phases, which included a one-on-one interview (Phase 1) and a web-based survey (Phase 2).

#### Phase 1

Phase 1 participants were patients recruited from an MG clinic at the University of South Florida to participate in a cross-sectional, qualitative, one-on-one interview study. The interviews were designed to better understand the patient experience with side effects of traditional treatments of MG and to understand the impact of these side effects on patient daily life and adherence to therapy. The participants in Phase 1 were recruited after being approached in person or over the telephone using a standardized screening script by clinic staff. Participants were required to: be at least 18 years of age at screening; have a physician-confirmed diagnosis of Class II, Class III, or Class IV MG as defined by the MGFA [6]; diagnosed at least 30 days prior to screening; currently receiving or have previously received drug therapy for MG; be able to speak, read, and write English sufficiently enough to complete study procedures; and be able to provide informed consent to participate in the study. The results of Phase 1 (transcribed recordings) were used to inform the development of a “focus prompt” – a statement asking patients to describe the side effects of MG and the impact of these side effects on daily life– administered in Step 1 of Phase 2. This focus prompt read: “Some of the treatments people with MG take have side effects. If you have ever experienced a side effect from your MG treatments, what side effects did you experience and how did that impact your life?”

#### Phase 2

Phase 2 participants were recruited from the MG patient registry, a project of the MGFA implemented and managed by the coordinating center at the University of Alabama Birmingham and the MGFA Registry Committee. The MGFA invited approximately 600 patients in the MG registry by email to participate in a web-based survey using group concept mapping (GCM) methods. The GCM approach is a two-step, mixed method approach that combines both qualitative and quantitative methodologies [7]. Participants were required to: be at least 18 years of age at screening; have a self-reported diagnosis of MG (i.e., patient responded “Yes” to the question: “Has your doctor diagnosed you with MG?” in the MGFA patient registry database); and be able to provide electronic consent to participate in the study.

In Step 1 of GCM, qualitative input was solicited via an online portal from study participants in a structured brainstorming/concept generation step that used a targeted focus prompt developed in Phase 1 of the study. Participants were asked to provide short statements in response to the focus prompt. Participants were encouraged to generate as many statements as possible. Upon completion of data collection, study investigators reviewed and synthesized participants’ raw responses to remove duplicate statements, eliminate irrelevant concepts, and correct grammar and syntax to ensure all statements were clear and easy to understand in preparation for the subsequent rating task (Step 2). Following the completion of the brainstorming/concept generation task, participants were asked to complete the 15-item Myasthenia Gravis Quality of Life questionnaire (MG-QOL15), the 8-item Morisky Medication Adherence Scale (MMAS-8), and a questionnaire to collect their sociodemographic and clinical information.

In Step 2 of GCM, the same group of participants was asked to return to the web portal to rate the synthesized responses in an unstructured process. The unstructured rating process allowed participants with different MG treatment histories to provide input based on their personal experiences. Specifically, participants were asked to rate a set of statements regarding side effects of treatments and impact on daily life. For the side effects statement set, participants were asked to rate the statements based on tolerability (1-Very tolerable to 4-Not at all tolerable) and severity of the side effects (1-Have not experienced this side effect to 4-Very severe). For the impact on daily life statement set, participants were asked to rate the statements based on frequency of impact (1-Never to 4-Almost always) and severity of impact (1-No impact to 4-Severe impact).

### Measures

#### Participant sociodemographic and clinical characteristics

For Phase 1, clinical staff from the recruiting institution completed a clinical form based on information documented in the participants’ medical records. The form collected background information pertinent to participants’ MG condition, including diagnosis, symptoms, and treatment history. For Phase 2, all participants completed a sociodemographic and clinical questionnaire to collect information on their age, gender, race, education, living situation, marital status, employment status, and MG clinical characteristics.

#### MG-QOL15

The MG-QOL15 is a brief 15-item, disease-specific, self-reported HRQOL measure derived from a 60-item MG-specific HRQOL scale [8–10]. Each item is rated on a scale of 0-Not at all to 4-Very much, and assesses the impact of MG on patients’ HRQOL as it relates to their physical, psychological, and social well-being and functioning. The reliability and validity of the MG-QOL15 has been previously demonstrated [9, 10].

#### MMAS-8

The MMAS-8 is an 8-item, self-reported questionnaire that assesses reasons for lack of adherence to medication treatment regimens including forgetting, carelessness, stopping the drug when feeling better, stopping the drug when feeling worse, forgetting due to travel, and not taking the drug due to inconvenience [11]. Response categories are yes/no for the first seven items, while the last item that assesses difficulty with remembering to take medications is rated on a 5-point scale ranging from 0-Never/rarely to 4-All the time. In this study, scoring for the MMAS-8 followed the developer’s scoring algorithm [11–13].^1^ The composite score for the MMAS-8 ranges from 0 to 8, with higher scores indicating better adherence. Scores can also be categorized into three levels of adherence: Low adherence (< 6), medium adherence (6 to < 8), high adherence (= 8). The concurrent and predictive validity of the MMAS-8 has been established [11].

### Statistical analysis

The interview data from Phase 1, in the form of the transcribed audio files, were systematically analyzed to evaluate participant feedback regarding side effects of MG and subsequent impact on daily life, using qualitative analysis software (ATLAS.ti, version 7.5.10). Descriptive statistics were used to characterize the study sample in Phases 1 and 2.

For the overall sample in Phase 2, mean (standard deviation [SD]) values were calculated for all side effect and daily life impact statements, and the distribution of ratings was examined. Post-hoc subgroup comparisons using analysis of variance (ANOVA) were also conducted to compare mean values for the side effect severity and tolerability ratings for patients that ever experienced the side effect, as well as the frequency and severity of impact on daily life statements by treatment refractory status. Specifically, participants were classified into three groups based on their self-reported responses to the medication questions asked in the MG clinical characteristics portion of the survey, including refractory (MG post-intervention status unchanged) to treatment and using intravenous immunoglobulin (IVIg), refractory but not using IVIg, and non-refractory to treatments for MG (Table 1).

**Table 1:** Refractory* status classifications for Phase 2 Post-hoc analysis

	Previous use of at least two immunosuppressive treatments	Current use of immunosuppressive treatment	Use of IVIg and/or plasmapheresis on more than four separate occasions over the past year
Refractory with IVIg	Yes	Yes	Yes
	Yes	No	Yes
	No	Yes	Yes
Refractory without IVIg	Yes	Yes	No
	Yes	No	No
Non-refractory	No	Yes	No

^*^Refractory = MG post-intervention status unchanged since initiation of treatment

IVIg, intravenous immunoglobulin

## Results

### Phase 1

A total of 14 individuals diagnosed with MG were interviewed between May and June of 2016. The mean (SD) age of the sample was 55.0 (±15.3) years and the majority were female (78.6%) and white (71.4%; Table 2). Almost all participants (92.9%) reported experiencing ocular symptoms, followed by symptoms in the neck, arms, and legs (85.7%). At the time of assessment, most patients (85.7%) were taking pyridostigmine for MG. The majority of the sample had low (42.9%) medication adherence levels according to the MMAS-8, approximately one-third had medium (35.7%) adherence and 21.4% had high adherence levels.

**Table 2:** Phases 1 and 2: Self-reported sociodemographic characteristics

Characteristic	Phase 1 Overall Sample	Phase 2 Overall Sample
	(*N* = 14)	(*N* = 242)
**Sex (female), n (%)**	11 (78.6%)	155 (64.0%)
**Age (years), mean (SD)[Range]**	55.0 (15.3) [21–79]	58.4 (13.4) [16–85]
Race^1^, n (%)		
White	10 (71.4)	224 (92.6)
Black or African American	3 (21.4)	9 (3.7)
Other	1 (7.1)	9 (3.7)
Ethnicity, n (%)		
Hispanic or Latino	0 (0)	7 (2.9)
Not Hispanic or Latino	14 (100)	235 (97.1)
Current living/domestic situation, n (%)		
Living alone	2 (14.3)	35 (14.5)
Living with a partner or spouse, family, or friends	12 (85.7)	202 (83.5)
Other	0 (0)	5 (2.1)
Employment Status^1^, n (%)^5^		
Employed, full-time	4 (28.6)	70 (28.9)
Employed, part-time	1 (7.1)	30 (12.4)
Homemaker	0 (0.0)	5 (2.1)
Student	2 (14.3)	4 (1.7)
Unemployed	0 (0.0)	7 (2.9)
Retired	4 (28.6)	86 (35.5)
Disabled	5 (35.7)	52 (21.5)
Other	1 (7.1)	11 (4.5)
Education^1^, n (%)		
Less than high school	0 (0.0)	2 (0.8)
Completed high school	3 (21.4)	13 (5.4)
Associate degree, technical or trade school	3 (21.4)	30 (12.4)
Some college	4 (28.6)	47 (19.4)
College	5 (35.7)	70 (28.9)
Graduate school	1 (7.1)	75 (31.0)
Other	0 (0.0)	5 (2.1)
Current Marital Status, n (%)		
Single	3 (21.4)	24 (9.9)
Married	7 (50.0)	173 (71.5)
Widowed	1 (7.1)	7 (2.9)
Separated	0 (0.0)	4 (1.7)
Divorced	2 (14.3)	27 (11.2)
Other	1 (7.1)	7 (2.9)

^1^Not mutually exclusive

All participants reported experiencing side effects from MG treatments, with the most commonly reported side effects being weight gain (*n* = 8, 57.1%), diarrhea/GI upset (*n* = 6, 42.9%), and headaches/migraines (*n* = 5, 35.7%). Overall, participants reported that side effects resulting from MG treatments had a significant impact on their daily life. The most common impacts included having to take additional medication to cope with side effects (*n* = 9, 64.3%), changing their diet (*n* = 6, 42.9%), having to buy new clothes due to weight gain (*n* = 6, 42.9%), general emotional impact (*n* = 6, 42.9%), and feeling bedridden/unable to leave the house (n = 6, 42.9%). Participants reported headaches/migraines (*n* = 5, 35.7%) and weight gain (n = 5, 35.7%) as the treatment side effects that affected their lives the most.

All 14 participants noticed some degree of change and improvement in their MG symptoms and/or function following commencement of treatment and considered the treatment to be working. In addition, all participants felt treatment was necessary to manage their MG symptoms and reported that efficacy/symptom improvement was the most important aspect when considering whether to continue with a treatment. Most participants (*n* = 10, 71.4%) reported that they never stopped taking or took less than the prescribed amount of MG medication on their own without consulting their doctor, while the remaining four participants (28.6%) reported doing so at least once.

### Phase 2

#### Step 1

**Fig. 1:**
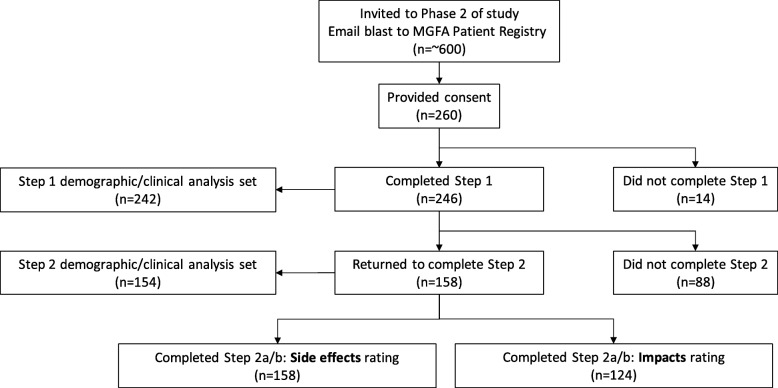
Phase 2 data disposition

The email request for participation was sent to MGFA registry patients in November 2016. Two hundred and sixty individuals (43.3% response rate) clicked on the study link and provided their consent to participate, of whom 246 (94.6%) completed the initial brainstorming/concept generation task (Step 1; Fig. 1). Of the 246, 242 participants (98.4%) also completed the sociodemographic and clinical questionnaires in Step 1. For this group, the mean (SD) age was 58.4 (±13.4) years with the majority being female (64.0%) and white (92.6%; Table 2).

Most of the 242 participants had experienced MG symptoms at some point in their eye muscles (90.1%), when chewing/swallowing/speaking (85.1%), in their neck, arms, and legs (81.8%), and with breathing (61.6%; Table 3). The majority (69.0%) reported taking pyridostigmine to treat their MG. One third (33.9%) of participants also reported currently taking some type of oral corticosteroids (e.g., prednisone). Slightly over one-third of the group (37.6%) had undergone a thymectomy. The most commonly reported comorbid conditions included: hypertension (33.9%), arthritis (24.0%), hypothyroidism (23.6%), and depression (21.1%). The mean (SD) MMAS-8 score was 6.3 (1.7) and the sample was evenly split between low (36.4%), medium (33.1%) and high (30.6%) medication adherence levels.

**Table 3:** Phase 2: Self-reported clinical characteristics

Characteristic	Overall Sample (*N* = 242) n (%)
In which of the following areas have you ever experienced myasthenia gravis symptoms?^1^	
Eye muscles	218 (90.1%)
Chewing, swallowing, speaking	206 (85.1%)
Neck, arm, and legs	198 (81.8%)
Breathing	149 (61.6%)
Other	42 (17.4%)
In the past month, where have you experienced myasthenia gravis symptoms?^1^	
Eye muscles	150 (62.0%)
Chewing, swallowing, speaking	139 (57.4%)
Neck, arm, and legs	137 (56.6%)
Breathing	87 (36.0%)
Other	50 (20.7%)
Current medication(s)/procedures for myasthenia gravis^1^	
Pyridostigmine	167 (69.0%)
Oral corticosteroids	82 (33.9%)
Mycophenolate mofetil	61 (25.2%)
Intravenous immunoglobulin (IVIg)	57 (23.6%)
Azathioprine	52 (21.5%)
Plasma exchange	14 (5.8%)
Rituximab	13 (5.4%)
Methotrexate	7 (2.9%)
Cyclophosphamide	1 (0.4%)
Tacrolimus	1 (0.4%)
Other	21 (8.7%)
None	15 (6.2%)
Medication(s) ever taken for myasthenia gravis^1^	
Oral corticosteroids	101 (41.7%)
Pyridostigmine	99 (40.9%)
Intravenous immunoglobulin (IVIg)	73 (30.2%)
Azathioprine	65 (26.9%)
Plasma exchange	57 (23.6%)
Mycophenolate mofetil	55 (22.7%)
Rituximab	15 (6.2%)
Cyclosporine A	11 (4.5%)
Methotrexate	7 (2.9%)
Neostigmine	6 (2.5%)
Cyclophosphamide	3 (1.2%)
Other	9 (3.7%)
None	41 (16.9%)
**Previous thymectomy (% yes)**	91 (37.6%)

^1^Not mutually exclusive

Based on the MG-QOL15 (data not presented), most participants’ HRQOL did not appear to be severely impacted by their MG, but most participants felt some frustration with their MG as indicated by their response to the item “*I am frustrated by my MG”* with 153 participants (63.2%) selecting “somewhat” or greater for this item.

A total of 874 individual, raw statements/concepts were collected across the 246 participants who completed Step 1. Following synthesis, a total of 35 statements related to side effects and 23 statements related to impacts on daily life were prepared for Step 2, where participants were invited back to the web-portal to rate the concepts.

#### Step 2

A total of 158 (64.2%) participants completed Step 2 (“returning sample”; Fig. 1). Eighty-eight participants did not return to the portal for Step 2 (“Step 1 only” group). All 158 completed the rating exercise for the side effect statements for Step 2, while 124 participants completed the rating exercise for the daily life impact statements.

No significant differences in demographic or clinical characteristics were found between the “Step 1 only” participants and “returning sample,” except fewer participants in the “returning sample” reported diabetes and depression compared with the “Step 1 only” group (12.3% vs. 25.0%, *p* = 0.012 and 16.2% vs. 29.5%, *p* = 0.015, respectively) (data not shown).

### Rating results

#### Side effect statements

Table 4 presents the severity and tolerability rating results of the 35 MG side effect statements for both the overall sample and for the subset of patients who had experienced the noted side effects. When considering all responses, the severity rating means for each statement ranged from 1.2 to 2.5, therefore most statements were rated somewhere between “have not experienced side effect” and “mildly severe”. The most severely rated side effect was weight gain, which had a mean (SD) of 2.5 (±1.1). This was closely followed by fatigue and diarrhea, which both had means (SD) of 2.4 (±1.0 to 1.1).

**Table 4:** Severity and tolerability of side effects of Myasthenia Gravis treatment (*N* = 158)^1^

Side Effect Statement	Overall Severity Sample^2^		Overall Tolerability Sample^3^		Patients who ever experienced side effect^4^			
					Severity^2^		Tolerability^3^	
	n	Mean (SD)	n	Mean (SD)	n	Mean (SD)	n	Mean (SD)
1. To gain weight	152	2.5 (1.1)	142	2.9 (1.0)	118	2.9 (0.8)	117	3.0 (0.9)
2. To have muscle weakness, causing my legs to be unsteady	151	2.2 (1.0)	136	2.7 (1.0)	102	2.7 (0.7)	99	2.8 (1.0)
3. To have low blood pressure	149	1.3 (0.8)	118	1.9 (1.0)	32	2.6 (0.8)	32	2.3 (1.0)
4. To lose weight	146	1.3 (0.7)	113	1.8 (1.0)	28	2.5 (0.7)	27	1.9 (0.9)
5. To have shortness of breath	145	2.0 (1.0)	124	2.7 (1.0)	88	2.7 (0.7)	83	3.0 (0.8)
6. Side effects such as acid reflux, heart burn, indigestion, esophageal ulcers, and irritable bowel syndrome	150	2.0 (1.1)	134	2.7 (1.0)	90	2.7 (0.8)	88	2.9 (0.8)
7. Severe side effects such as: aseptic meningitis, sepsis, pneumonia, pleural edema, fluid in the heart, allergic/anaphylactic reactions, or internal bleeding.	149	1.5 (1.0)	119	2.6 (1.4)	32	3.3 (0.7)	32	3.3 (1.0)
8. Changes to my skin	150	2.1 (1.0)	133	2.5 (1.0)	94	2.7 (0.7)	94	2.7 (0.8)
9. To have headaches or migraines	149	1.9 (1.0)	129	2.5 (1.1)	79	2.6 (0.8)	79	2.8 (0.9)
10. Muscle wasting	148	1.7 (0.8)	122	2.6 (1.2)	66	2.5 (0.6)	65	2.8 (0.9)
11. Vision changes, such as double vision	148	2.1 (1.1)	127	2.7 (1.0)	88	2.8 (0.8)	88	2.9 (0.9)
12. Watery eyes, increased saliva, nasal drainage, secretions, or mucus	149	2.1 (1.0)	134	2.4 (0.9)	101	2.7 (0.7)	99	2.6 (0.8)
13. To be fatigued. I felt weak, tired, drained	149	2.4 (1.0)	128	2.8 (0.9)	116	2.8 (0.7)	107	3.0 (0.8)
14. Side effects such as constipation, stomach cramps, intestinal pain and cramping, stomach bloating, and gas	148	2.2 (1.0)	134	2.7 (0.9)	106	2.7 (0.7)	104	2.8 (0.8)
15. To become forgetful, confused, and have problems concentrating	147	1.8 (0.9)	122	2.6 (1.0)	79	2.6 (0.7)	78	2.8 (0.9)
16. To have mood changes	148	2.1 (1.0)	130	2.7 (1.1)	95	2.7 (0.8)	94	2.9 (1.0)
17. To have fluid retention	146	1.7 (0.9)	122	2.5 (1.0)	66	2.6 (0.7)	65	2.7 (0.9)
18. To have heart problems	147	1.6 (0.9)	121	2.5 (1.2)	53	2.6 (0.8)	51	2.9 (1.0)
19. Changes to my laboratory values (blood tests)	149	1.9 (0.9)	129	2.3 (1.0)	87	2.5 (0.7)	85	2.5 (0.9)
20. My extremities (toes, fingers) to become tingly and sensitive to the cold	146	1.6 (0.8)	122	2.3 (1.0)	65	2.4 (0.7)	65	2.7 (0.8)
21. A mild allergic reaction such as hives or rashes	145	1.4 (0.7)	117	2.1 (1.0)	46	2.3 (0.6)	46	2.4 (0.8)
22. To have diarrhea	148	2.4 (1.1)	133	2.7 (1.0)	107	2.9 (0.8)	104	2.9 (0.9)
23. My face to bloat or swell	147	2.0 (1.0)	132	2.5 (1.0)	85	2.7 (0.7)	82	2.7 (0.9)
24. Feel lightheaded or dizzy	145	1.7 (0.8)	124	2.4 (1.0)	73	2.4 (0.6)	72	2.7 (0.7)
25. To become depressed, anxious, or nervous	146	2.0 (1.0)	126	2.6 (1.0)	85	2.7 (0.8)	84	2.9 (0.9)
26. Decreased my immune response	148	2.1 (1.0)	133	2.8 (1.0)	95	2.7 (0.8)	91	3.1 (0.7)
27. To develop other medical conditions	145	1.6 (0.9)	129	2.7 (1.3)	53	2.7 (0.8)	54	3.1 (1.0)
28. To become infertile	144	1.0 (0.3)	108	2.0 (1.3)	3	2.7 (1.2)	4	2.5 (1.3)
29. To have muscle cramps in my legs	150	2.2 (0.9)	137	2.6 (0.9)	113	2.6 (0.8)	111	2.7 (0.8)
30. Aches and pain in various parts of my body	146	1.9 (0.9)	125	2.4 (0.9)	86	2.6 (0.7)	83	2.7 (0.8)
31. To have nausea and/or vomiting.	146	1.6 (0.9)	119	2.4 (1.1)	61	2.5 (0.7)	60	2.8 (0.7)
32. Muscle twitching and eye twitching or eyelid drooping.	149	2.0 (1.0)	131	2.5 (0.9)	93	2.6 (0.8)	92	2.7 (0.7)
33. To have hot flashes and to sweat profusely	149	1.8 (0.9)	124	2.4 (1.0)	80	2.5 (0.7)	77	2.6 (0.8)
34. Develop blood clots	144	1.2 (0.7)	112	2.4 (1.4)	11	3.5 (0.8)	11	3.9 (0.3)
35. High blood pressure	143	1.5 (0.8)	121	2.3 (1.2)	48	2.5 (0.7)	50	2.7 (1.0)

^1^Four participants did not complete the demographic section in Part 1, but completed Part 2

^2^Severity was assessed on a 1–4 scale, with 1 being “have not experienced this side effect” and 4 being “very severe”

^3^Tolerability was assessed on a 1–4 scale, with 1 being “very tolerable” and 4 being “not at all tolerable”

^4^Patients who rated side effect as a “2-Mildly severe” or higher

Due to the large percentage of participants responding with a 1 (“Never”) on many of the side effect rating statements, the analysis was also conducted omitting the ratings of participants who had not experienced the listed side effects (Table 4). These mean ratings were generally more severe and ranged between 2.3 (for mild allergic reactions) to 3.5 (for development of blood clots), with the majority ranging between 2.5 and 2.7, corresponding to moderately severe symptoms. The most severely rated side effects were those relating to development of blood clots and other serious conditions (e.g., aseptic meningitis, sepsis, and pneumonia), weight gain, and diarrhea. The means scores for these side effect statements ranged from 2.9 (weight gain and diarrhea) to 3.5 (blood clots).

The tolerability rating results of the 35 side-effect statements were similar to the severity ratings (Table 4). For the overall sample, the means for each statement ranged from 1.8 to 2.9, indicating that, on average, most statements were rated somewhere between “tolerable” and “somewhat tolerable”. When only the ratings of participants who had previously experienced the listed side effects were examined, mean tolerability ratings were slightly higher (more severe). The side-effect statements rated as the least tolerable and most severe included the more serious side effects such as: blood clots, aseptic meningitis, sepsis, pneumonia, pleural edema, fluid in the heart, allergic/anaphylactic reactions, or internal bleeding. Weight gain, decreased immunity, fatigue, shortness of breath, and development of other medical conditions were additional side effects that were poorly tolerated. 

#### Impact on daily life statements

Table 5 presents the severity and frequency rating results for the 23 statements related to the impact of side effects on daily life. The severity rating means for each statement ranged from 1.7 to 2.5, meaning that most impacts were rated somewhere between “no impact” and “mild impact”. This finding was consistent with the mean MG-QOL15 scores. The most severe impacts were related to sleep interference, reduction in physical activity, and reduction in social activities (each had severity ratings of either 2.4 or 2.5). The least severe impacts were related to healthcare utilization issues (i.e., requiring additional medical procedures, being hospitalized). For more than half of the statements in the impact severity rating exercise, participants responded with the lowest possible rating (“No impact”), which likely reflects that many patients did not experience certain impacts.

**Table 5:** Severity and frequency of impacts on daily life due to side effects of myasthenia gravis treatment (*N* = 124)

Impact Statement	Severity^1^		Frequency^2^	
	n	Mean (SD)	n	Mean (SD)
1. To become depressed	121	2.1 (1.0)	123	2.0 (0.9)
2. Affected my ability to work or attend school	118	2.3 (1.1)	122	2.2 (1.1)
3. Made me moody	122	2.1 (1.0)	123	2.1 (0.9)
4. Caused me to sleep more than usual	121	1.8 (0.9)	120	1.8 (1.0)
5. Caused me to discontinue or avoid taking my medications	119	1.8 (1.1)	123	1.8 (1.0)
6. Caused insomnia or interferes with my sleep. I am not able to get a good night’s sleep	122	2.4 (1.1)	124	2.4 (1.1)
7. Caused me to require additional medical procedures	122	1.7 (1.1)	124	1.5 (0.9)
8. Interfered with my ability to care for my family	120	2.0 (1.0)	122	2.0 (1.1)
9. Made me very frustrated and/or demoralized	121	2.1 (1.0)	122	2.1 (1.0)
10. Caused me to visit the emergency room and/or be hospitalized	119	1.9 (1.2)	122	1.5 (0.8)
11. Sometimes made my MG symptoms worse	121	2.0 (1.0)	121	1.7 (0.8)
12. Have limited my daily activities	120	2.3 (1.0)	123	2.2 (1.1)
13. Have limited me physically	121	2.5 (1.1)	123	2.5 (1.1)
14. Made me very irritable and short-tempered	122	1.9 (1.0)	123	1.9 (1.0)
15. With the MG treatment that I’m prescribed it is difficult to adjust the correct dose that I need to control my MG symptoms	122	1.7 (0.9)	123	1.7 (0.9)
16. Have made me feel very self-conscious, ugly, and/or unattractive	122	1.9 (1.1)	123	2.0 (1.1)
17. Made me uncomfortable being around other people	120	2.0 (1.0)	119	1.9 (1.0)
18. Made me worry about catching infections from other people so I reduced my social activities	119	2.4 (1.0)	122	2.4 (1.1)
19. Have limited my mobility	121	2.2 (1.1)	123	2.2 (1.1)
20. Have caused me to be so tired, I avoid leaving the house or going out in public	120	2.0 (1.0)	122	2.0 (1.0)
21. Decreased my quality of life	119	2.3 (1.1)	123	2.3 (1.1)
22. Side effects sometimes caused me to have to choose between tolerating my MG symptoms or the side effects of medication	122	2.0 (1.1)	122	2.0 (1.1)
23. Made life very stressful and/or overwhelming	119	1.9 (1.0)	123	2.0 (1.1)

^1^Severity was assessed on a 1–4 scale, with 1 being “no impact” and 4 being “severe impact”

^2^Frequency was assessed on a 1–4 scale, with 1 being “never” and 4 being “almost always”

A majority of the impact statements were rated somewhere between “never” and “rarely” (Table 5). The most frequent impacts (i.e., those with the highest means) were related to sleep interference, reduction in physical activity, and reduction in social activities (each had frequency ratings of either 2.4 or 2.5).

#### Refractory subgroup analysis

For the refractory subgroup analysis, 41 participants were classified as “Refractory with IVIg”, 88 participants were “Refractory without IVIg”, and 24 participants were “Non-Refractory” (Table 6). There were no significant differences in demographics or self-reported clinical characteristics between the groups (data not shown), except that the Refractory with IVIg group had a greater proportion of participants self-reporting to be disabled compared to the Refractory without IVIg and Non-refractory samples (39.0% vs. 17.0% vs. 20.8%, respectively; *p* = 0.022), and the Refractory with IVIg sample appeared to have a greater number of individuals experiencing MG symptoms associated with breathing (82.9% vs. 63.6 and 29.2%, *p* < 0.001).

**Table 6:** Severity^1^ of side effects of myasthenia gravis treatment^2^ by refractory group

Side Effect Statement	Refractory with IVIg (*N* = 41), n, mean (SD)	Refractory without IVIg (*N* = 88), n, mean (SD)	Non-Refractory (*N* = 24) n, mean (SD)	*P* value^3^
1. …caused me to gain weight.	32, 3.2 (0.7)	69, 2.8 (0.8)	14, 2.6 (0.9)	0.068
2. …caused me to have muscle weakness, causing my legs to be unsteady.	30, 2.9 (0.8)	58, 2.7 (0.6)	12, 2.6 (0.5)	0.173
3. …caused me to have low blood pressure (hypotension).	8, 2.8 (0.9)	21, 2.7 (0.7)	3, 2.0 (0.0)	0.317
4. …caused me to lose weight.	8, 2.8 (1.0)	17, 2.5 (0.6)	2, 2.0 (0.0)	0.455
5. …caused me to have shortness of breath.	24, 2.7 (0.8)	51, 2.8 (0.7)	11, 2.2 (0.4)	0.051
6. …caused side effects such as acid reflux, heart burn, indigestion, esophageal ulcers, and irritable bowel syndrome.	23, 2.5 (0.7)	51, 2.9 (0.8)	15, 2.7 (0.9)	0.213
7. …caused severe side effects such as: aseptic meningitis, sepsis, pneumonia, pleural edema, fluid in the heart, allergic/anaphylactic reactions, or internal bleeding.	12, 3.4 (0.8)	16, 3.2 (0.7)	3, 3.0 (0.0)	0.551
8. …caused changes to my skin, such as bruising more easily, taking longer for wounds to heal, acne, stretch marks, and scalp burn.	25, 2.8 (0.7)	56, 2.7 (0.7)	10, 2.6 (0.5)	0.823
9. …caused me to have headaches or migraines.	28, 2.6 (0.8)	44, 2.6 (0.8)	5, 2.2 (0.4)	0.483
10. …caused muscle wasting.	20, 2.5 (0.7)	38, 2.4 (0.6)	6, 2.3 (0.5)	0.856
11. …caused vision changes, such as double vision.	26, 2.8 (0.8)	50, 2.9 (0.8)	10, 2.5 (0.7)	0.413
12. …caused watery eyes, increased saliva, nasal drainage, secretions, or mucus.	25, 2.6 (0.6)	56, 2.7 (0.7)	16, 2.7 (0.8)	0.717
13. …caused me to be fatigued. I felt weak, tired, drained, and exhausted while on this treatment.	36, 2.8 (0.7)	64, 2.9 (0.7)	14, 2.6 (0.6)	0.228
14. …caused side effects such as constipation, stomach cramps, intestinal pain and cramping, stomach bloating, and gas.	28, 2.7 (0.7)	64, 2.7 (0.8)	12, 2.8 (0.9)	0.960
15. …caused me to become forgetful, confused, and have problems concentrating.	23, 2.5 (0.5)	43, 2.7 (0.7)	10, 2.3 (0.5)	0.241
16. …caused me to have mood changes, which may include mood swings, anger or rage, hyperactivity, or lack of interest.	26, 2.8 (0.8)	56, 2.7 (0.8)	11, 2.4 (0.5)	0.292
17. …caused me to have fluid retention (such as edema or swelling) in my arms or legs.	22, 2.6 (0.7)	36, 2.5 (0.7)	6, 2.8 (1.0)	0.624
18. …caused me to have heart problems such as an increased heart rate, heart palpitations, or an irregular heartbeat.	20, 2.7 (0.7)	29, 2.6 (0.8)	2, 2.5 (0.7)	0.944
19. …caused changes to my laboratory values (blood tests) such as white blood cells, red blood cells, calcium, vitamin B12, creatinine, altered kidney function, or increased cholesterol.	30, 2.5 (0.7)	51, 2.5 (0.7)	5, 2.4 (0.5)	0.885
20. …caused my extremities (toes, fingers) to become tingly and sensitive to the cold.	21, 2.3 (0.6)	32, 2.4 (0.6)	11, 2.6 (0.8)	0.367
21. …caused a mild allergic reaction such as hives or rashes.	18, 2.3 (0.6)	22, 2.4 (0.7)	5, 2.0 (0.0)	0.374
22. …caused me to have diarrhea.	30, 2.7 (0.8)	61, 3.0 (0.8)	14, 3.0 (0.9)	0.436
23. …caused my face to bloat or swell.	28, 2.7 (0.7)	46, 2.7 (0.7)	8, 2.8 (1.0)	0.951
24. …made me feel lightheaded or dizzy.	26, 2.3 (0.6)	37, 2.4 (0.6)	8, 2.5 (0.8)	0.823
25. …caused me to become depressed, anxious, or nervous.	25, 2.7 (0.7)	49, 2.8 (0.8)	n, 2.4 (0.5)	0.318
26. …decreased my immune response causing me to become sick more easily.	29, 2.7 (0.7)	56, 2.8 (0.8)	7, 2.4 (0.5)	0.428
27. …caused me to develop other medical conditions such as Cushing’s syndrome, osteoporosis, diabetes, cancer, cataracts or macular degeneration, adrenal insufficiency, and lipomatosis.	20, 2.7 (0.7)	28, 2.8 (0.8)	4, 2.5 (0.6)	0.625
28. …caused me to become infertile.	0	3, 2.7 (1.2)	0	
29. …caused me to have muscle cramps in my legs.	32, 2.5 (0.8)	61, 2.7 (0.7)	18, 2.4 (0.8)	0.343
30. …caused me aches and pain in various parts of my body.	29, 2.4 (0.6)	46, 2.7 (0.7)	9, 2.6 (0.7)	0.110
31. …caused me to have nausea and/or vomiting.	20, 2.4 (0.6)	35, 2.6 (0.7)	5, 2.8 (0.8)	0.318
32. …caused muscle twitching and eye twitching or eyelid drooping.	24, 2.6 (0.7)	54, 2.6 (0.8)	13, 2.7 (0.9)	0.919
33. …caused me to have hot flashes and to sweat profusely.	25, 2.6 (0.8)	43, 2.5 (0.7)	10, 2.4 (0.5)	0.594
34. …caused me to develop blood clots.	5, 3.0 (1.0)	6, 3.8 (0.4)	0	0.093
35. …caused me to have high blood pressure (hypertension).	19, 2.4 (0.6)	26, 2.6 (0.8)	3, 2.3 (0.6)	0.579

^1^ Severity was assessed on a 1–4 scale, with 1 being ‘have not experienced this side effect’ and 4 being ‘very severe.’ Participants who endorsed an item with ‘1–have not experienced this side effect’ were excluded from the analysis

^2^ Five participants were excluded from this analysis. Four participants did not complete the demographic section, but completed Step 2. One participant completed the demographic section, but did not complete Step 2

^3^ *P*-values to compare the three groups are based on ANOVA for continuous variables

When severity rating results were compared for participants who had previously experienced each of the listed side effects by refractory treatment status (Refractory with IVIg vs. Refractory without IVIg vs. Non-refractory), no statistically significant differences were observed between groups for any of the listed side effects. However, the Refractory with IVIg group generally reported greater side-effect severity, followed by the Refractory without IVIg group and the non-refractory group (Table 6).

Among participants who had previously experienced each of the listed side effects, the tolerability ratings of the side-effect statements were generally higher (i.e., poorer tolerability) for the two refractory groups compared to the non-refractory group, although these differences were only statistically significant for the following (data not shown): *“caused me to gain weight”,* “*caused me to have fluid retention (such as edema or swelling) in my arms or legs”,* and *“caused me to have high blood pressure (hypertension)”*.

In examining the frequency of impact ratings of the daily life impact statements, statistically significant differences between refractory groups were seen for the majority of the 23 statements (Table 7). The Refractory with IVIg group tended to have the highest means (i.e., greatest frequency of impact) while the Non-refractory group tended to have the lowest means, thereby indicating that the Refractory with IVIg group had the greatest impact from their MG treatment. Similar results were evidenced for the severity of daily life impact ratings, where the Refractory with IVIg group had the highest means; all but 6 comparisons were statistically significantly different between groups (data not shown).

**Table 7:** Frequency^1^ of impact on daily life of myasthenia gravis treatment^2^ by refractory group

Impact Statement	Refractory with IVIg (*n* = 30) mean (SD)	Refractory without IVIg (*n* = 71) mean (SD)	Non-Refractory (*n* = 20) mean (SD)	*P* value^3^
1. To become depressed.	2.3 (0.8)	2.0 (0.9)	1.7 (0.7)	0.036
2. Affected my ability to work or attend school.	2.9 (1.0)	2.2 (1.1)	1.6 (0.9)	<.001
3. Made me moody.	2.5 (1.0)	2.1 (0.9)	1.6 (0.7)	0.001
4. Caused me to sleep more than usual.	2.2 (1.0)	1.7 (1.0)	1.6 (0.8)	0.063
5. Caused me to discontinue or avoid taking my medications.	1.9 (1.1)	1.8 (1.0)	1.6 (1.1)	0.520
6. Caused insomnia or interferes with my sleep. I am not able to get a good night’s sleep.	2.8 (1.0)	2.5 (1.1)	1.8 (1.0)	0.008
7. Caused me to require additional medical procedures.	1.8 (1.1)	1.5 (0.8)	1.3 (0.8)	0.083
8. Interfered with my ability to care for my family.	2.6 (1.1)	2.0 (1.0)	1.4 (0.7)	<.001
9. Made me very frustrated and/or demoralized.	2.6 (0.9)	2.1 (1.0)	1.6 (0.9)	0.004
10. Caused me to visit the emergency room and/or be hospitalized.	2.0 (0.9)	1.5 (0.8)	1.1 (0.2)	<.001
11. Sometimes made my MG symptoms worse.	1.9 (0.8)	1.7 (0.8)	1.5 (0.7)	0.185
12. Have limited my daily activities.	2.7 (1.1)	2.2 (1.0)	1.6 (0.9)	0.002
13. Have limited me physically.	3.0 (1.0)	2.4 (1.2)	1.9 (1.0)	0.001
14. Made me very irritable and short-tempered.	2.3 (0.9)	1.9 (1.0)	1.4 (0.7)	0.004
15. With the MG treatment that I’m prescribed it is difficult to adjust the correct dose that I need to control my MG symptoms.	2.0 (1.1)	1.7 (0.9)	1.5 (0.8)	0.109
16. Have made me feel very self-conscious, ugly, and/or unattractive.	2.3 (1.2)	1.9 (1.1)	1.6 (0.9)	0.043
17. Made me uncomfortable being around other people.	2.4 (1.0)	1.9 (1.0)	1.4 (0.7)	0.006
18. Made me worry about catching infections from other people.	2.5 (1.1)	2.5 (1.0)	1.7 (1.2)	0.006
19. Have limited my mobility,	2.7 (1.1)	2.2 (1.1)	1.6 (1.0)	0.002
20. Have caused me to be so tired, I avoid leaving the house or going out in public.	2.5 (1.0)	2.0 (1.0)	1.6 (0.9)	0.006
21. Decreased my quality of life.	2.8 (1.0)	2.3 (1.0)	1.6 (0.9)	<.001
22. Side effects sometimes caused me to have to choose between tolerating my MG symptoms or the side effects of medication.	2.3 (1.1)	1.9 (1.0)	1.6 (1.0)	0.059
23. Made life very stressful and/or overwhelming.	2.6 (1.0)	1.9 (1.1)	1.5 (0.7)	<.001

^1^ Frequency was assessed on a 1–4 scale, with 1 being ‘never’ and 4 being ‘almost always’

^2^ Three participants were excluded from this analysis because they did not complete the questions assessing refractory status and could not be classified

^3^ P-values to compare the three groups are based on ANOVA for continuous variables

## Discussion

Qualitative interviews conducted among the 14 patients with MG in Phase 1 of this study revealed that the most prevalent and impactful side effects resulting from MG treatment(s) were weight gain, diarrhea/GI upset, and headaches/migraines. Overall, all 14 participants reported that side effects resulting from MG treatments had a significant impact on their daily life. All of the participants felt that treatment was necessary to manage their MG symptoms and all reported that efficacy/symptom improvement was the most important aspect when considering whether to continue with a treatment [14, 15]. A recent cross-sectional study of HRQOL in two large population MG cohorts found the same HRQOL levels across time [16, 17]. This was despite more MG treatment options and better diagnostics of MG subgroups becoming available over the study period. Boldingh et al. 2015 also provided a summary table of previous studies conducted using the HRQOL 36-item Short Form Health Survey (SF-36) among MG patients and presented the study outcomes compared to population norms [16]. All previous studies reported diminished HRQOL compared to the healthy control groups [14].

This current study builds upon the previous work by describing patient experiences with side effects of traditional treatments of MG as described by patients, thus, furthering the understanding of the impact of MG therapy side effects on patients’ daily life and HRQOL using two sources of information, including patient input as collected via the MGQOL-15 and through an open-ended exercise where patients indicate side effects and impacts they themselves experience. Results from the work reported here demonstrate that, while many of the side effects and daily life impacts were not endorsed by patients considered non-refractory (as expected), side effects and daily life impacts related to weight gain and fatigue were particularly concerning to patients with MG. Furthermore, results also highlight that sleep interference, decline in physical activity, and reduction in social activities pose the greatest impacts on MG patients’ daily life.

The mean ratings for the daily life impact statements in Phase 2 should be interpreted with some caution as there were many patients in this study who experienced minimal side effects and, therefore, likely had minimal negative daily life impacts. Even participants who experienced few side effects were still asked to rate the impact of their side effects on their daily life. It is plausible to assume these participants generally responded with the floor response of “never” and “no impact,” thereby diluting the frequency and severity ratings for the daily life impact statements. In addition, a general limitation of this study is that the MGFA registry population is a self-selected group of patients, which may limit the generalizability of the results.

Upon running the subgroup analysis, which compared results stratified by participants’ refractory treatment status, it was determined that most side effects tended to be more severe and/or less tolerable among the Refractory with IVIg group compared to the other two groups. The Non-refractory group tended to have the lowest rating scores for the side effect concepts, as well as for the daily life impact statements. Despite this, when severity and tolerability of side effects were compared among participants with previous experience of the listed side effects, there were no statistically significant differences observed between groups for any of the listed side effects. This suggests that regardless of MG severity or treatment refractory status, side effects of treatment pose a similar burden to patients in terms of severity and tolerability, however these results should be interpreted with caution given the small number of non-refractory patients with side effects in this analysis. In terms of daily life impact, the differences between the groups were statistically significant for the frequency and severity of impact ratings for the majority of the 23 daily life impact statements. These results are not surprising given that the Refractory with IVIg group is likely to be the subgroup with the most advanced, severe MG and these patients may have tried the greatest number of treatments, but with limited success.

This study did not explore whether weight gain, fatigue, or the aforementioned HRQOL impacts had a direct effect on adherence to treatment; although, this would be an important area to cover in future research. This study was not designed to elucidate which MG treatments (e.g., pyridostigmine, oral corticosteroids) were responsible for certain side effects, or the relationship between MG type, duration of illness, and the type of treatments utilized; however, these topics may be of interest in future research to explore the relationship between classes of treatment, MG type, and side effects and daily life impact. It is also important to note that it is often difficult for patients to separate side effects and impacts resulting from their treatment, from symptoms and symptom impacts resulting from the disease of interest or even from their comorbid conditions. Although participants in this study were instructed to think about and rate issues resulting from their MG treatment, it is possible that their ratings were influenced by the severity of their MG symptoms. Additionally, once a medication causing side effects has been stopped, participants may not accurately recall the burden or impact of those side effects. In addition, the items used in the rating task did not undergo validation analyses, however the study process and software have been previously validated [18].

## Conclusions

This study has confirmed the serious burden of side effects resulting from traditional MG treatments and the negative impact of side effects on patient daily life. It is important to understand how side effects impact patients as this may have repercussions on patient treatment adherence and ultimately on their health outcomes. The qualitative and quantitative findings from this study provide evidence of the burden of treatment side effects experienced by patients with MG, especially those refractory to treatment. Based on these findings, there is a need for more effective and more tolerable treatment options for patient with MG.

## Data Availability

The data are not available in a public repository, as complex analytics were required to compile the data in a way that is in alignment with the original research objectives. However, study authors will consider sharing the data, by request, on a case by case basis.

## Abbreviations

ANOVA: Analysis of variance
FDA: Food and Drug Administration
GCM: Group concept mapping
GI: Gastrointestinal
HRQoL: Health-related quality of life
IVIg: Intravenous immunoglobulin
MG: Myasthenia gravis
MGFA: Myasthenia Gravis Foundation of America
MG-QOL15: 15-item Myasthenia Gravis Quality of Life questionnaire
MMAS-8: 8-item Morisky Medication Adherence Scale
SD: Standard deviation
SF-36: 36-item Short Form Health Survey
Term: Definition
US: United States

## Footnotes

1 The MMAS (8-item) content, names and trademarks are protected by US copyright and trademark laws. Permission for use of the scale and its coding is required. A license agreement is available from: Donald E. Morisky, ScD, ScM, MSPH, Professor, Department of Community Health Sciences, UCLA School of Public Health, 650 Charles E. Young Drive South, Los Angeles, CA 90095–1772.©- 2006 Donald E. Morisky. All right reserved. MMAS, Morisky Medication Adherence Scale, and Morisky are trademarks of Donald E. Morisky and may be used only with permission. v.05. dmorisky@ucla.edu
